# Ship Trim Optimization: Assessment of Influence of Trim on Resistance of MOERI Container Ship 

**DOI:** 10.1155/2014/603695

**Published:** 2014-01-20

**Authors:** Salma Sherbaz, Wenyang Duan

**Affiliations:** College of Shipbuilding Engineering, Harbin Engineering University, Harbin, Heilongjiang 150001, China

## Abstract

Environmental issues and rising fuel prices necessitate better energy efficiency in all sectors. Shipping industry is a stakeholder in environmental issues. Shipping industry is responsible for approximately 3% of global CO_2_ emissions, 14-15% of global NO_*X*_ emissions, and 16% of global SO_*X*_ emissions. Ship trim optimization has gained enormous momentum in recent years being an effective operational measure for better energy efficiency to reduce emissions. Ship trim optimization analysis has traditionally been done through tow-tank testing for a specific hullform. Computational techniques are increasingly popular in ship hydrodynamics applications. The purpose of this study is to present MOERI container ship (KCS) hull trim optimization by employing computational methods. KCS hull total resistances and trim and sinkage computed values, in even keel condition, are compared with experimental values and found in reasonable agreement. The agreement validates that mesh, boundary conditions, and solution techniques are correct. The same mesh, boundary conditions, and solution techniques are used to obtain resistance values in different trim conditions at Fn = 0.2274. Based on attained results, optimum trim is suggested. This research serves as foundation for employing computational techniques for ship trim optimization.

## 1. Introduction

Global temperature increase by 2°C above preindustrial level is expected to cause severe devastation on global scale. The only way to overcome this issue is to reduce global greenhouse gas (GHG) emissions. Intergovernmental Panel on Climate Change [1] concluded that global greenhouse gas (GHG) emissions need to be 50–85% below current levels in 2050 to reach this target. However, all IPCC scenarios indicate significant increase in global GHG emissions by 2050 which is alarming [2]. Thus, all stakeholders responsible for GHG emissions face a great challenge to achieve this target.

**Figure 1:**
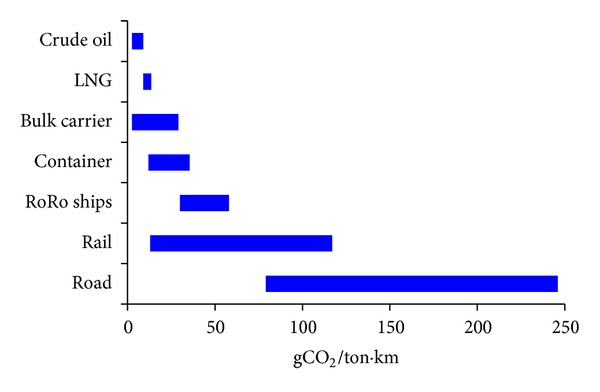
Ship CO_2_ emissions comparison to rail and road.

Shipping industry is one of the stakeholders in environmental issues. Shipping industry is responsible for approximately 3% of global CO_2_ emissions, 14-15% of global NO_*X*_ emissions, and 16% of global SO_*X*_ emissions [3, 4]. Shipping industry also has a critical role in global economy. Intercontinental trade, bulk transport of raw materials, and import/export of affordable food and goods are carried out through ships. It is estimated that almost 90% of the world trade goods are carried by ships. For the most part, this trade has little or no alternative means of transportation other than ships at this point and foreseeable future. Furthermore, shipping is a better environmental option for transportation compared to other available means due to lowest gCO_2_/ton·km emissions as shown in Figure 1.

**Figure 2:**
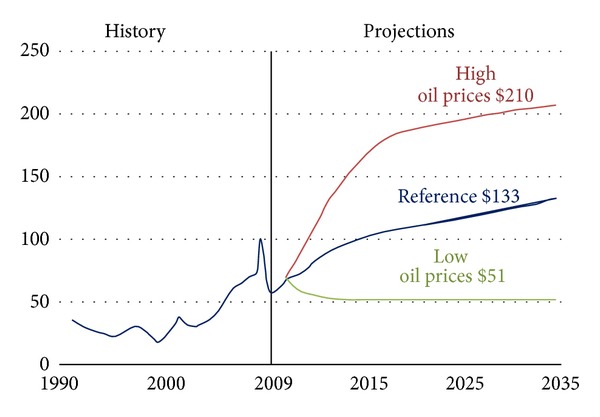
World fuel prices ($ per barrel).

Future scenarios indicate that if preventive measures are not taken, CO_2_ emissions from ships will more than double by 2050 [5]. European Union (EU) and United Nations Framework Convention on Climate Change (UNFCCC) are pushing hard to regulate global emissions. International Maritime Organization (IMO) is currently working to establish GHG regulations for international shipping with technical, operational, and market-based policy instruments. It seems clear that IMO regulations pertaining to CO_2_ emissions from shipping will continue to expand in coming years. Furthermore, fuel prices have ever been rising and expected to continue rising in future as shown in Figure 2 [6]. High fuel prices in the long run are also an incentive to focus on new ways for better energy effectiveness in addition to better environmental performance.

Ship trim optimization has gained enormous momentum in recent years being an effective operational measure for better energy efficiency to reduce emissions. Ship trim optimization principles are based on the fact that ship experiences different (more or less) resistance through water for the same speed and draft depending on its trim. Ship trim optimization simply advocates selecting the trim condition for minimum resistance. The resistance through water is then proportional to the amount of required power and governs the required fuel burn rate. Hence, less resistance implies fewer emissions and more economical operation by fuel savings.

**Figure 3:**
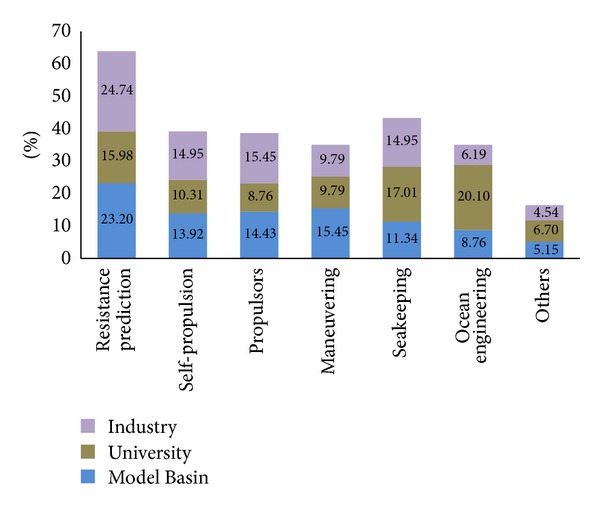
Applications of CFD in marine hydrodynamics.

Computational techniques are increasingly popular for design and optimization applications in all engineering disciplines. Computational fluid dynamics (CFD) simulation of complex phenomenon is now possible owing to increase in computational power and parallel processing. CFD simulation techniques are increasingly being used in ship hydrodynamics applications as shown in Figure 3 [7]. The purpose of this study is to present CFD simulation methodology for ship trim optimization problem. The trim optimization study of MOERI container ship (KCS) hull by employing CFD techniques has been presented as model case. The mesh, boundary conditions, and solution techniques are validated by comparing computed values to experimental values in even keel condition. Same mesh, boundary conditions, and solution techniques are later employed to obtain resistance values in different trim conditions at Froude number corresponding to design speed. Based on attained results, optimum trim condition at design speed is suggested. This research serves as foundation for employing CFD techniques to ship trim optimization problem.

## 2. Ship Model

**Figure 4:**
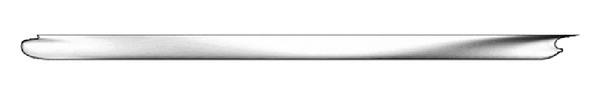
MOERI container ship (KCS) hull.

MOERI container ship (KCS) hull has been selected as a model case, shown in Figure 4. The main reason for using this hull is that extensive model test data exists for resistance at different Froude numbers. KCS hullform was conceived as a model hullform for a modern container ship with a bulbous bow. KCS underwent extensive model testing/experimental exploration to provide baseline experimental data to enhance understanding of flow, physics and CFD validation for container ship design applications. The Korea Research Institute for Ships and Ocean Engineering (KRISO) now named Maritime and Ocean Engineering Research Institute (MOERI) performed towing tank experiments to obtain data for resistance, mean flow and free surface waves applicable to design and CFD validation purposes. KCS is one of the three hullforms explored in Gothenburg 2010 workshop on numerical ship hydrodynamics. The other hullforms explored in this workshop are Korean VLCC designed at MOERI (KVLCC2) and US combatant model (DTMB 5415). The main parameters of the model, used in the present study, are summarized in Table 1.

## 3. Numerical Method

SHIPFLOW code is used in global approach to simulate the flow around ship hull. Potential flow module XPAN of SHIPFLOW is employed to calculate wave making resistance using nonlinear free surface boundary conditions. Viscous flow calculations are carried out using viscous flow module XCHAP of SHIPFLOW software. Three-dimensional, incompressible, two-phase (air and water) flow is considered. Steady state computations are performed. Steady state computations are justified due to the fact that ship design convention deals with ship trim optimization problem based on calm water tow-tank testing.

The governing equations, that is, continuity and Navier-Stokes equation, are given by

(1) $$\begin{array}{c} \frac{\partial u_{i}}{\partial x_{i}}=0,\\ \frac{\partial }{\partial x_{j}}\left(\overset{-}{u_{ı}}\;\overset{-}{u_{ȷ}}\right)=-\frac{1}{\rho }\frac{\partial \overset{-}{p}}{{\partial x}_{i}}+F_{i}+\frac{1}{\rho }\frac{\partial }{{\partial x}_{j}}\left({\overset{-}{\sigma }}_{ji}+R_{ji}\right), \end{array}$$

where $\overset{-}{u_{ı}}$, $\overset{-}{u_{ȷ}}$, $\overset{-}{p}$, and ${\overset{-}{\sigma }}_{ji}$ are time-averaged velocity components (*i*, *j* = 1,2, 3), pressure, and stress, respectively. *ρ* is fluid density, *F*_*i*_ is body force, and Reynolds stress $R_{ji}=-\rho \overset{-}{{\overset{´}{u}}_{ı}{\overset{´}{u}}_{ȷ}}$ is given by

(2) $$\begin{array}{c} \rho \overset{-}{{\overset{´}{u}}_{ı}}\;\overset{-}{{\overset{´}{u}}_{ȷ}}=-\mu_{t}\left(\frac{{\partial u}_{i}}{{\partial x}_{j}}+\frac{{\partial u}_{j}}{{\partial x}_{i}}\right)+\frac{2}{3}\rho k\delta_{ij}. \end{array}$$

The turbulence model SST *k*-*ω* is used to close above equations [8]. This model is believed to be one of the best choices to simulate turbulent flow around ship hull [9]. The model *k*-*ω*-SST is a hybrid model which employs *k*-*ω* model near the wall and a *k*-*ϵ* model, transformed to resemble a *k*-*ω* model, outside of near the wall region. The coefficients and additional cross-diffusion term from the transformed *k*-*ϵ* model are combined in the intermediate region by using blending functions. The equations for *k* and *ω* in the SST model are written to be [10]

(3) ∂k∂t+∂(ujk)∂xj  =−u´ı− u´ȷ−∂ui∂xj−β∗kω+∂∂xj((υ+σkυT)∂k∂xj),

(4) ∂ω∂t+∂(ujω)∂xj=−γυTu´ı− u´ȷ−∂ui∂xj−β∗ω2+∂∂xj((υ+σωυT)∂ω∂xj)+2σω21−F1ω∂k∂xj∂ω∂xj,

where *F*_1_ is blending function for combining *ω* and *ϵ*-equations. The blending of *ω* and *ϵ*-terms is performed in the wake region of the boundary layer.

## 4. Grid Generation and Boundary Conditions

**Figure 5:**
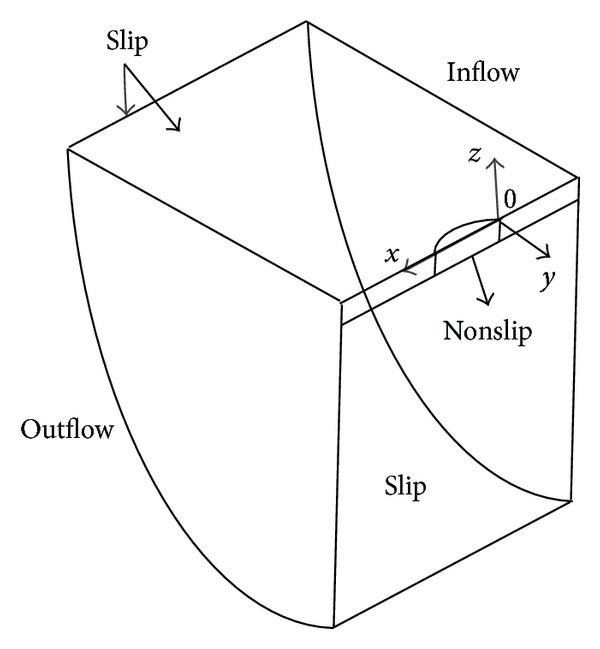
Computational domain around hull.

The panel generator XMESH is employed for generating paneling and free surface on the body for potential flow calculations. The total numbers of panels generated by XMESH are 11908. The H-O grid topology is employed using XGRID for viscous flow computations. The computational domain consists of inflow plane, outflow plane, outer boundary, hull surface, and symmetry plane as shown in Figure 5. The inflow and outflow planes are at a distance of 0.5L_PP_ front and behind the hull. The domain outer boundary lengthens 2.0L_PP_ from the hull surface. The number of mesh elements generated by XGRID is 1.051 M.

The inflow boundary conditions are design velocity, *k*, *ω*, and zero pressure gradient, whereas outflow boundary conditions are zero gradient of velocity, *k*, *ω*, and fixed pressure. The slip condition used at the outer boundary implies that normal velocity component and normal gradient of all other flow quantities are zero. The nonslip condition used at the symmetry plane (*y* = 0) and flat free surface (*z* = 0) entails that velocity components, *k*, and pressure gradient are zero. The Roe scheme is employed for discretization of connective terms when the finite volume Reynolds-Averaged Navier-Stokes (RANS) solver XCHAP is used in conjunction of *k*-*ω* SST turbulence model. The central differences are employed for discretization of remaining terms.

## 5. Resistance Calculations in Even Keel Condition

**Figure 6:**
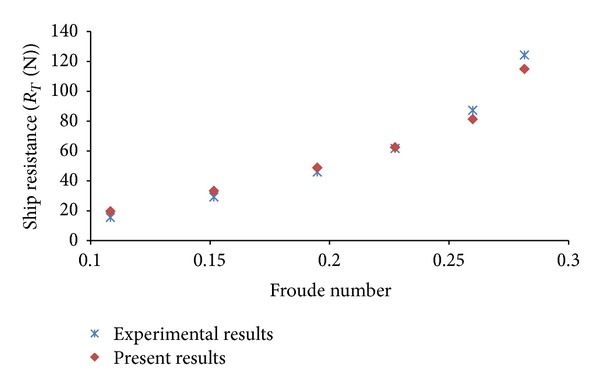
Comparison of computed and experimental results.

**Figure 7:**
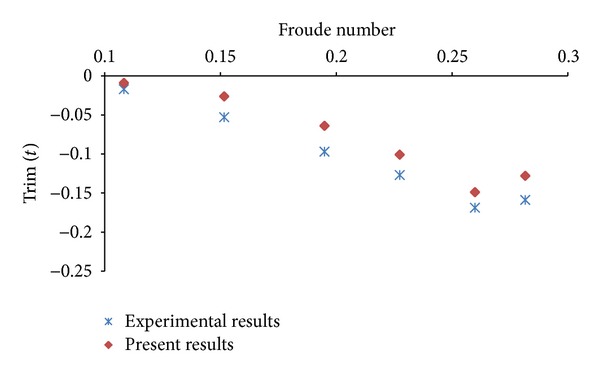
Comparison of simulation and experimental results of trim (*t*).

**Figure 8:**
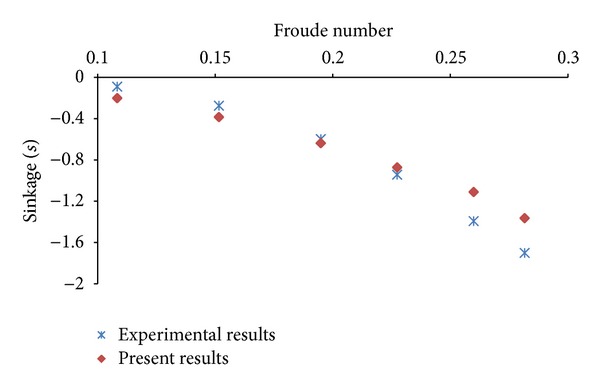
Comparison of computed and experimental results of sinkage (*s*).

The model test data is available for resistance of various ship hullforms in even keel condition. In the present study, first total resistance of KCS hullform is calculated at different Froude numbers in even keel condition to validate mesh, boundary conditions, and solution techniques. The computed values of total resistance, trim, and sinkage, in even keel condition, are compared with experimental values [11] and found in reasonable agreement. The plots pertaining to comparison of computed and experimental results are exhibited as Figures 6, 7, and 8. The same mesh, boundary conditions, and solution techniques are later used to calculate resistance in different trim conditions.

## 6. Resistance Calculations in Different Trim Conditions

**Figure 9:**
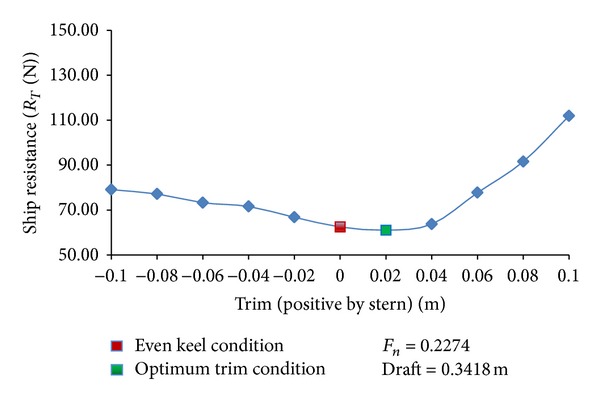
KCS hull trim optimization plot.

The validated mesh, boundary conditions, and solution techniques are employed to calculate KCS hull resistance values at design draft 0.3418 m in different trim conditions. The trim values range from 0.02 m to 0.1 m for stern trim and 0.02 m to 0.1 m for bow trim in steps of 0.02 m. The Froude number is 0.2274 corresponding to design speed of full scale ship. The resistance versus trim plot for KCS hull is shown in Figure 9. Comparing with even keel condition, significant increase in total resistance has been noticed when ship was trimmed by bow. On trimming the ship by stern, total resistance of the hull reduced at first and increased later. The optimum trim point is 0.02 m trim by stern.

**Figure 10:**
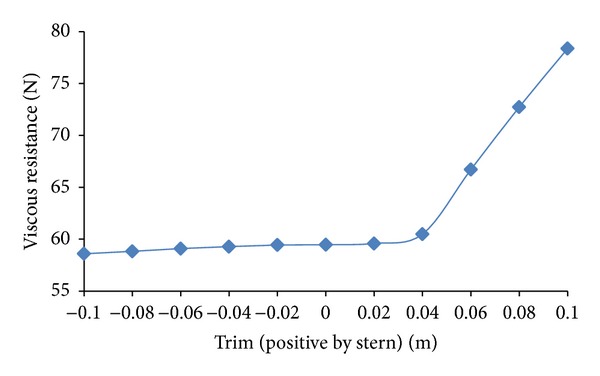
Viscous resistance as a function of trim.

**Figure 11:**
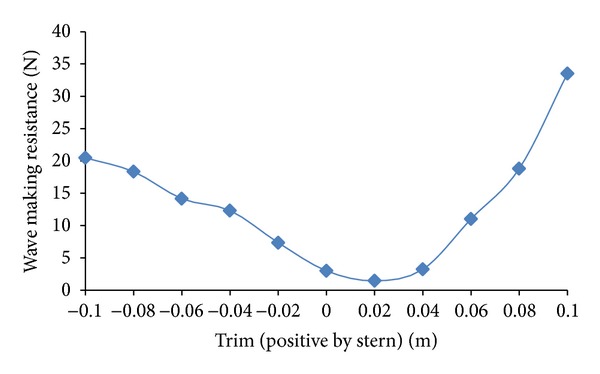
Wave making resistance as a function of trim.

**Figure 12:**
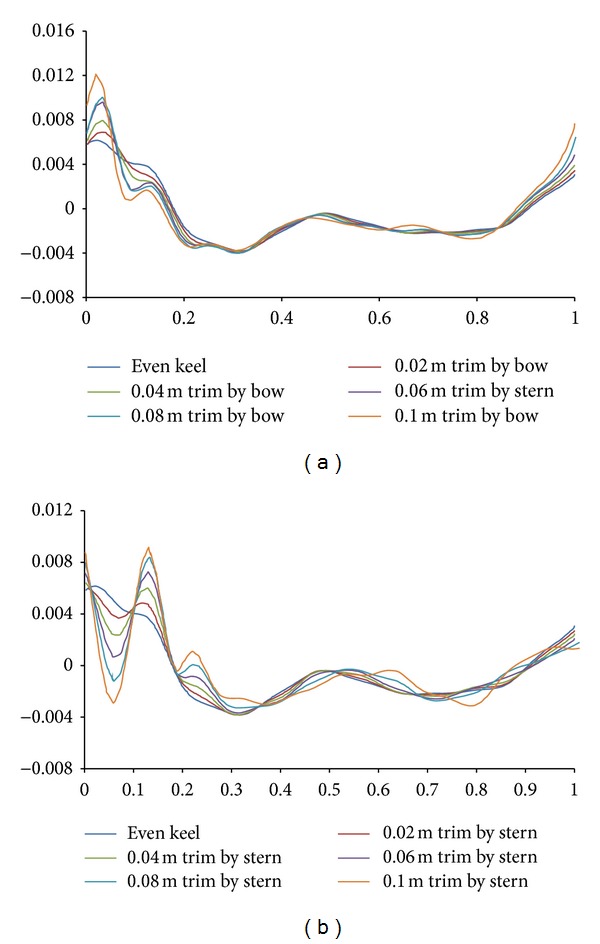
Predicted wave profile at Fn = 0.2274 in different trim conditions.

The total resistance is broken down into viscous resistance and wave making resistance to analyze the effect of trim on each part. Viscous resistance changes slightly with change in trim as shown in Figure 10. The phenomenon supports the fact that there is slight change in underwater water area with change in trim, and viscous resistance manifests accordingly being a function of underwater area. The effect of trim is dominant on wave making resistance part as shown in Figure 11. The percentage-wise change in wave making resistance *R*_*W*_, viscous resistance *R*_*V*_, and total resistance *R*_*T*_ due to trim at Fn = 0.2274 is summarized in Table 2. The wave making performance of hull is complex and can only be analyzed by detailed towing tank experimentation or computational studies similar to the one being presented in this paper. The plots pertaining to comparison of wave profiles in even keel condition and different trim conditions are presented in Figure 12.

The trim can have a significant impact on ship resistance; the extent of trim effects will be different for different hullforms. Ship trim optimization analysis must be performed for all in-service and under design hullforms.

## 7. Conclusions

Ship trim optimization has gained enormous momentum in recent years being an effective operational measure for better energy efficiency to reduce emissions. Ship trim optimization analysis has traditionally been done through tow-tank testing for a specific hullform. Computational techniques are increasingly popular for design and optimization applications in all engineering disciplines. The advantages of using computation techniques over experimental model testing are fast turnaround time during design phase and lesser cost. CFD simulation techniques are being increasingly used in ship hydrodynamics applications.

In the present study, CFD simulation techniques have been deployed for MOERI container ship (KCS) hull trim optimization. KCS hull parameters, in even keel condition, at different Froude numbers are attained by computational techniques and compared to experimental values to validate mesh, boundary conditions, and solution techniques. These validated mesh, boundary conditions, and solution techniques are employed to calculate KCS hull resistance values in different trim conditions.

The results showed that trim has pronounced increasing effect on resistance during bow trim. The effect on resistance is varying during stern trim and optimum trim point is 0.02 m trim by stern. The study of viscous and wave making components reveals that viscous resistance changes slightly with change in trim whereas trim has a dominant effect on wave making resistance.

It is pertinent to highlight that optimum trim be achieved by shifting weights while avoiding taking ballast water, since increased displacement due to ballast water can cause higher resistance and thus higher fuel consumption. Ship trim optimization has enormous potential in liquid cargo carriers where it is easy to manipulate trim by shifting liquid weight.

## Figures and Tables

**Table 1:** Main characteristics of ship model.

Parameters	Dimensions
Length (m)	7.3570
Beam (m)	1.0190
Draft (m)	0.3418
Wetted surface area (m^2^)	9.5441
Block coefficient	0.6510

**Table 2:** Percentage-wise change in wave making and viscous and total resistance due to trim.

Trim	**−0.1**	**−0.08**	**−0.06**	**−0.04**	**−0.02**	**0**	**0.02**	**0.04**	**0.06**	**0.08**	**0.1**

Δ*R*_W_ (%)	85.39	83.69	78.87	75.72	59.29	0.00	−51.84	7.14	72.87	84.10	91.08
Δ*R*_V_ (%)	−1.47	−1.07	−0.62	−0.30	−0.05	0.00	0.20	1.69	10.85	18.23	24.11
Δ*R*_T_ (%)	21.01	19.04	14.73	12.77	6.45	0.00	−2.29	1.95	19.63	31.77	44.18

## References

[1] Bernstein L, Bosch P, Canziani O (2007). *Climate Change 2007: An Assessment of Intergovernmental Panel on Climate Change*.

[2] Solomon S, Qin D, Manning M (2007). *Fourth Assessment Report of Intergovernmental Panel on Climate Change: The AR4 Synthesis Report*.

[3] Buhaug Ø, Corbett JJ, Endresen Ø (2009). *Second IMO GHG Study*.

[4] Endresen Ø, Sørgård E, Behrens HL, Brett PO, Isaksen ISA (2007). A historical reconstruction of ships’ fuel consumption and emissions. *Journal of Geophysical Research D*.

[5] Eyring V, Kohler HW, Lauer A, Lemper B (2005). Emissions from international shipping: 2. Impact of future technologies on scenarios until 2050. *Journal of Geophysical Research*.

[6] Doman LE (2010). *International Energy Outlook Report*.

[7] Campana EF, Hino T, Carrica P The specialist committee on computational fluid dynamics: Final Report and Recommendations to the 26th ITTC.

[8] Menter FR (1994). Two-equation eddy-viscosity turbulence models for engineering applications. *AIAA Journal*.

[9] Zhang Z-R, Liu H, Zhu S-P, Zhao F (2006). Application of CFD in ship engineering design practice and ship hydrodynamics. *Journal of Hydrodynamics*.

[10] Flowtech International AB (2007). *XCHAP Theoretical Manual*.

[11] Larsson L, Stern F, Visonneau M (2010). *A Workshop on Numerical Ship Hydrodynamics Proceedings*.

